# Dose-dense regimen versus conventional three-weekly paclitaxel combination with carboplatin chemotherapy in first-line ovarian cancer treatment: a systematic review and meta-analysis

**DOI:** 10.1186/s13048-023-01216-z

**Published:** 2023-07-10

**Authors:** Wenjian Gong, Ruidi Yu, Canhui Cao, Yong Fang, Xuejiao Zhao, Qinglei Gao

**Affiliations:** 1 Cancer Biology Research Center (Key Laboratory of the Ministry of Education), Tongji Hospital, Tongji Medical College, Huazhong University of Science and Technology, Wuhan, 430000 People’s Republic of China; 2 National Clinical Research Center for Obstetrics and Gynecology, Department of Gynecological Oncology, Tongji Hospital, Tongji Medical College, Huazhong University of Science and Technology, Wuhan, Hubei, 430030 China

**Keywords:** Ovarian cancer, Paclitaxel, Carboplatin, Dose-dense regimen

## Abstract

**Background:**

Paclitaxel dose-dense regimen has been controversial in clinical trials in recent years. This systematic review and meta-analysis tried to evaluate the efficacy and safety of paclitaxel dose-dense chemotherapy in primary epithelial ovarian cancer.

**Methods:**

An electronic search following PRISMA guidelines was conducted (Prospero registration number: CRD42020187622), and then a systematic review and meta-analysis of included literature were initiated to determine which regimen was better.

**Results:**

Four randomized controlled trials were included in the qualitative evaluation, and 3699 ovarian cancer patients were included in the meta-analysis. The meta-analysis revealed that the dose-dense regimen could prolong PFS (HR0.88, 95%CI 0.81–0.96; *p* = 0.002) and OS (HR0.90, 95%CI 0.81–1.02; *p* = 0.09), but it also increased the overall toxicity (OR = 1.102, 95%CI 0.864–1.405; *p* = 0.433), especially toxicity of anemia (OR = 1.924, 95%CI 1.548–2.391; *p* < 0.001), neutropenia (OR = 2.372, 95%CI 1.674–3.361; *p* < 0.001). Subgroup analysis indicated that the dose-dense regimen could significantly prolong not only PFS (HR0.76, 95%CI 0.63–0.92; *p* = 0.005 VS HR0.91, 95%CI 0.83–1.00; *p* = 0.046) but also OS (HR0.75, 95%CI 0.557–0.98; *p* = 0.037 VS HR0.94, 95%CI 0.83–1.07; *p* = 0.371) in Asian, and overall toxicity was significantly increased in Asians (OR = 1.28, 95%CI: 0.877–1.858, *p* = 0.202) compared to non-Asians (OR = 1.02, 95%CI 0.737–1.396, *p* = 0.929).

**Conclusion:**

Paclitaxel dose-dense regimen could prolong PFS and OS, but it also increased the overall toxicity. Therapeutic benefits and toxicity of dose-dense are more obvious in Asians compared to non-Asians, which need to be further confirmed in clinical trials.

**Supplementary Information:**

The online version contains supplementary material available at 10.1186/s13048-023-01216-z.

## Background

Ovarian cancer is women’s seventh most common cancer and the most lethal gynecological malignancy. Most ovarian cancer patients are at an advanced stage by the time of diagnosis due to the lack of better early screening, and the 5-year overall survival rate is about 30%. According to data from the American Cancer Society medical information, there were about 21,410 new cases of ovarian cancer and 13,770 deaths in the United States in 2021 [1], the 5-year overall survival rate of ovarian cancer has not significantly improved in the past decade [2].

The standard treatment for advanced ovarian cancer is primary debulking surgery followed by chemotherapy. Paclitaxel combined with carboplatin is the first-line chemotherapy for ovarian cancer [3]. The conventional three-weekly therapy consisted of intravenous infusion of paclitaxel 175 mg/m^2^ over 3 h and carboplatin area under the curve (AUC) 5 or AUC 6, repeated every three weeks for 6 cycles. The dose-dense regimen is paclitaxel 80 mg/m^2^ intravenous infusion for 1 h given on days 1, 8, and 15, carboplatin AUC 5- AUC 6 given on day 1 of a three weeks cycle, for a total of 6 cycles, or paclitaxel 80 mg/m^2^ intravenous infusion plus carboplatin AUC2 once a week, for a total of 18 cycles. The dose-dense regimen paclitaxel at 80 mg/m^2^ was recommended because it was a feasible and reasonably well-tolerated regimen [4]. In the past decade, there has been great interest in dose-dense therapy deemed to be a new attempt to improve the survival outcomes of breast cancer, and those clinical trials revealed that dose-dense regimen had overall survival advantages [5, 6]. At the same time, several large-scale randomized controlled trials (RCT) in dose-dense regimen for ovarian cancer had been implemented, but the results in efficacy and safety were conflicting, so the conventional three-weekly regimen is still as preferred regimen, while the dose-dense regimen is recommended as other regimens, or used only in certain situations.

Therefore, as the largest RCT (ICON8) recently reported the results [7], we systematically reviewed the RCTs of paclitaxel dose-dense therapy for ovarian cancer in recent years, explored the principle of dose-dense therapy and discussed the reasons for the controversies in these RCT results. And a meta-analysis was performed to compare the efficacy in terms of survival outcomes and toxicity to determine which regimen is the best for ovarian cancer.

## Methods

### Search strategy

This study was registered at the International Prospective Register of Systematic Reviews (PROSPERO, CRD42020187622). We searched PubMed and Medline for eligible studies published up to August, 2021. The search followed Preferred Reporting Items for Systematic reviews and Meta–Analyses (PRISMA) guidelines [8]. Search terms used were “ovarian cancer OR ovarian tumor OR ovarian Neoplasms” AND “Platinum OR Cisplatin OR carboplatin” AND “Paclitaxel OR Taxol” AND “Dose-dense OR Weekly OR Once a week”. There were no other restrictions, including language. The eligible study designs were randomized controlled trials, while case reports, comments, and letters were not included because the data provided by the authors were insufficient.

The titles and abstracts from the search results were reviewed independently by two authors (Wenjian Gong and Ruidi Yu) to determine their relevance to our study question. Any disagreement was resolved by discussion with the third author (Canhui Cao).

All of the included articles were screened by including criteria as follows: (1) patients were those newly diagnosed with epithelial ovarian cancer, fallopian tube cancer or primary peritoneal cancer by histology or cytology; (2) Patients received weekly dose-dense paclitaxel combined with platinum regimen chemotherapy, and the control group received standard paclitaxel three-weekly chemotherapy. Articles would be excluded if following the scenario occurs: (1) Non-randomized controlled study; (2) Phase II clinical research; (3) Valid data were not extractable.

### Data extraction and endpoint

The following variables were extracted from eligible studies: abbreviation of RCT, author name and publication time, region, sample size, chemotherapy method and drug dosage. Progression-free survival (PFS) and overall survival (OS) are the primary endpoints of this study, and adverse reactions greater than three grades such as neutropenia, anemia, and vomiting are the second endpoints. For studies that cannot extract PFS and OS, Engauge Digitizer11.0 was used to extract data from the survival curve [9].

### Statistical analysis

We performed a meta-analysis, where pooled HR (hazard ratio) was calculated with a 95% CI to explore risk factors for PFS and OS, pooled OR (odds ratio) was calculated with a 95% CI to explore risk factors for adverse reactions. A formal Q statistical test and I^2^ statistical tests were performed to evaluate the heterogeneity of each study, then either the Mantel–Haenszel fixed-effects model or the DerSimonian-Laird random-effects model was used to calculate the pooled OR with a 95% CI based on heterogeneity results. A *P*-value < 0.10 and I^2^ > 50% was considered as an indicator for random-effects model, with the alternative being a fixed-effect model [10]. In addition, funnel plot and Egger’s test were used to analyze publication bias. All aforementioned statistical analyses were performed using STATA version 12.0 software (STATA Corporation, College Station, TX, USA).

## Results

### Search results

**Fig. 1:**
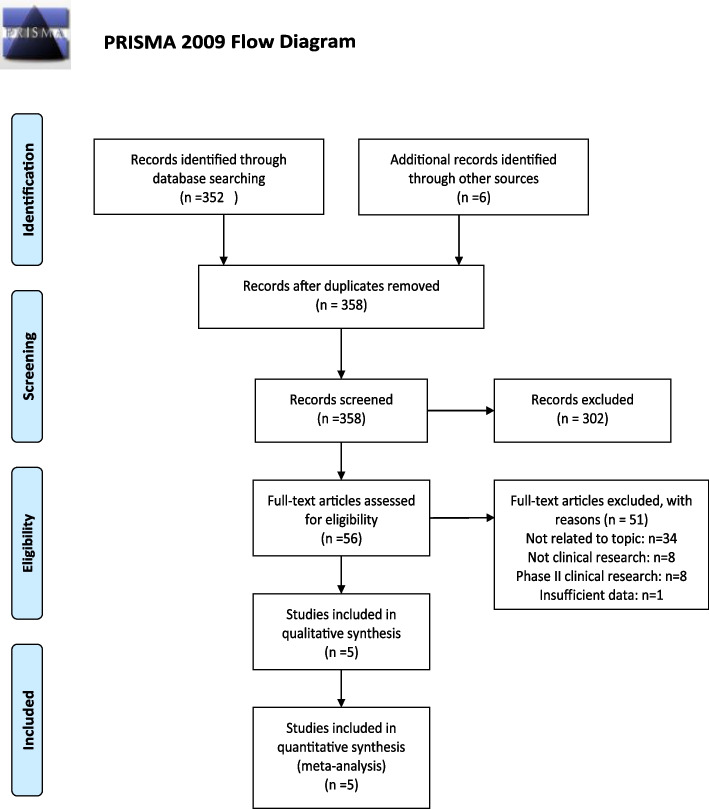
PRISMA flowchart of included studies for systematic review

A total of 358 papers were searched. After the article type was limited to randomized controlled trial, 56 papers were remaining. 34 papers were excluded because they were not related to our research topic, 8 were descriptive research or analytical research, 8 were phase II clinical trials, and 1 had no suitable data extraction, therefore, 5 papers were ultimately included [7, 11–14] (Fig. 1). A total of 4 randomized controlled studies were included, the characteristics and details of included randomized controlled trials were depicted in Table 1, and all of the articles were issued between 2001 and 2021. A total of 3699 patients with ovarian cancer were included in the study.

**Table 1:** Summary of basic characteristics of included trials evaluating dose-dense regimen vs. conventional three-week paclitaxel chemotherapy in epithelial ovarian cancer

Study	Country	FIGO stage	Number of patients (Weekly vs Every 3 weeks)	Chemotherapy regimen	Median PFS	OS
JGOG3016 [13] 2013	Japan	II–IV EOC	631(312: 319)	1: q3W P 180 mg/m^2^ + q3W C AUC 6 2: q1W P 80 mg/m^2^ + q3W C AUC6	28.2 m vs. 17.5 m (HR = 0.76; *p* = 0.0037)	Median OS: 100.5 m vs. 62.2 m (HR = 0.79; *p* = 0.039)
MITO-7 [11] 2014	Italy, France	IC–IV	810(409:404)	1: q3W P 175 mg/m^2^ + q3W AUC 6 2: q1W P 60 mg/m^2^ + q1W C AUC 2	17.3 m vs. 18.3 m (HR = 0.96; *p* = 0.66)	2 years OS: 78.9% vs. 77.3% (HR = 1.2; *p* = 0.22)
GOG 0262 [10] 2016	US, Canada, South Korea	II-IV	692(346:346)	1: q1W P 80 mg/m^2^ + q3W C AUC6 2: q3W P 175 mg/m^2^ + q3W C AUC 6	Not receive bevacizumab: 14.2 m vs. 10.3 m (HR = 0.62; *p* = 0.03)	
ICON8 [6] 2019	European	IC-IV	1566 (522: 522:522)	1: q3W P 175 mg/m^2^ + q3W C AUC 5–6 2: q1W P 80 mg/m^2^ + q3W C AUC 5–6 3: q1W P 80 mg/m^2^ + q1W C AUC 2	17.7 m vs. 20.8 m vs. 21.0 m (HR = 0·9 – arms 2 vs. 1) (HR = 0.93 – arms 3 vs. 1)	Median OS^a^: HR = 0·90(0.74–1.1) – arms 2 vs 1 HR = 0.88(0.71–1.07) – arms 3 vs 1

*FIGO* International Federation of Gynecology and Obstetrics, *PFS* progression-free survival, *OS* overall survival, *EOC* epithelial ovarian carcinoma, *P* paclitaxel, *C* carboplatin, *AUC* area under the curve (unit: mg/mL/min), *W* week

^a^ Median OS for ICON8 was extracted by Engauge Digitizer11.0 from the survival curve

### The dose-dense regimen was recommended

In JGOG3016 study, 637 patients with stage II to IV epithelial ovarian cancer, fallopian tube cancer, or primary peritoneal cancer were randomized to receive a dose-dense regimen or conventional three-weekly therapy. In the dose-dense group, the median PFS significantly increased by 11 months (28.2 months vs 17.5 months, HR 0.76, 95% CI 0.62- 0.91; *p* = 0.0037), and median OS had 38 months prolongation (100.5 months vs 62.2 months, HR 0.79, 95% CI 0.63–0.99; *p* = 0.039). Dose-dense treatment caused more grade 3 and 4 anemia (69% vs 44%), but other toxic effects were similar.

Dose-dense treatment showed both excellent tolerability and promising efficacy for ovarian cancer. According to subgroup analysis, patients with residual disease more than 1 cm or serous histological subtypes had a significant improvement in median PFS (17.6 months vs 12.1 months, HR 0.71, 95% CI 0.56–0.89; *p* = 0.0029; 28.7 months vs 17.5 months, HR 0.70, 95% CI 0.57–0.86; *p* = 0.0007) and median OS (51.2 months vs 33.5 months, HR 0.75, 95% CI 0.57–0.97; *p* = 0.0027; 100.5 months vs 61.2 months, HR 0.76, 95% CI 0.59–0.97; *p* = 0.0252). Histopathological analysis of the patients in the study found that patients with mesenchymal transition (MT) type receiving dose-dense regimen had a significantly better median PFS (1.8 vs 1.2 years, *p* = 0.01) [15]. Three other experiments also showed that dose-dense treatment improved survival time compared with conventional three-weekly treatment [16–18].

MITO-5 trial revealed that dose-dense treatment had a significantly lower frequency of toxicity profile and great tolerance in elderly ovarian cancer patients over 70 years old with multiple tumor complications and functional impairment [19].

In GOG-0262 study, 692 patients were prospectively stratified according to whether they were elected to receive bevacizumab, and then they were randomly assigned to receive a dose-dense regimen or conventional three-weekly therapy. The overall results showed that the PFS of dose-dense regimen group was similar to that of conventional three-weekly therapy group (14.7 vs. 14.0 months; HR0.89; 95% CI 0.74–1.06; *p* = 0.18). And there was no significant extension of PFS in patients receiving bevacizumab who received paclitaxel weekly compared with every three weeks (14.9 vs. 14.7 months; HR0.99; 95% CI 0.83–1.20; *p* = 0.60). It was worth noting that among patients who did not receive bevacizumab, dose-dense regimen had 3.9 months prolongation in PFS than conventional three-weekly therapy (14.2 vs. 10.3 months; HR0.62; 95% CI 0.40–0.95; *p* = 0.03) [11].

MITO-7 trial included 822 ovarian cancer patients, they randomly accepted conventional three-weekly therapy or dose-dense regimen (paclitaxel 60 mg/m^2^ plus carboplatin AUC 2 once a week). The median PFS of the dose-dense regimen group and the conventional three-weekly therapy group were 18.3 months and 17.3 months respectively (HR 0.96; *p* = 0.66). The two-year OS rate was 77.3% vs 78.9% (HR 1.20; *p* = 0.22). Quality-of-life scores revealed that the dose-dense regimen group was superior to conventional 3-weekly therapy group (treatment-by-time interaction; *p* < 0.0001). And dose-dense regimen group had fewer toxicity profiles: grade 3/4 neutropenia (42.0% vs 50.0%; *p* = 0.021), febrile neutropenia (0.5% vs 3.0%; *p* = 0.01), grade 3/4 thrombocytopenia (1.0% vs 7.0%; *p* < 0.0001), and grade 2 or worse neuropathy (6.0% vs 17.0%; *p* < 0.0001) [12].

These studies demonstrated that dose-dense regimen might be a reasonable alternative regimen in patients with advanced ovarian cancer, which could bring therapeutic benefits, such as prolonged survival time, fewer toxic effects, and better quality of life. However, several other randomized controlled trials had no significant evidence that dose-dense regimen could replace conventional three-weekly therapy.

### Conventional three-weekly therapy was recommended

The ICON8 trial included 1566 ovarian cancer patients who were predominantly European population, they were randomly assigned to three groups. Group 1 received conventional 3-weekly therapy, group 2 and group 3 received dose-dense regimen. It was found that there was no PFS benefit in either dose-dense regimen. The PFS of conventional three-weekly therapy was 24.4 months, while the PFS of the dose-dense regimen was 24.9 months respectively (HR = 0.92, 95%Cl 0.77–1.09; *p* = 0.45) and 25.3 months (HR = 0.94, 95% Cl 0.79–1.12; *p* = 0.56), sensory neuropathy and febrile neutropenia incidences were similar across groups. And patients who received a conventional three-weekly regimen had a better quality of life. Therefore, they concluded that dose-dense regimen was not recommended as a first-line treatment option for non-Japanese women with newly diagnosed ovarian cancer [7, 20].

Three other trials revealed the same conclusion as ICON8: dose-dense regimen had not improved benefit in terms of OS, PFS or RR, but had a better toxicity profile, therefore it might not be an appropriate alternative to the conventional three-weekly therapy as first-line treatment [21–23].

### Meta-analysis of progression-free survival and overall survival

Therefore, these trials could be divided into two groups by their conclusions, group 1 showed that dose-dense regimen significantly prolonged PFS and OS, however, group 2 indicated that dose-dense regimen did not have adequate benefit in PFS and OS, but could reduce toxicity profile. A meta-analysis of these trials was performed, results verified the conclusion. It is worth mentioning that, in dose-dense regimen, carboplatin was used either every three weeks or once a week, so we divided the ICON8 study into ICON8-1(q3W P 175 mg/m^2^ + q3W C AUC 5–6 vs q1W P 80 mg/ m^2^ + q3W C AUC 5–6) and ICON8-2(q3W P 175 mg/ m^2^ + q3W C AUC 5–6 vs q1W P 80 mg/ m^2^ + q1W C AUC 2). In MITO7, carboplatin was administered once a week (q1W C AUC 2). In GOG 0316 and GOG 0262, carboplatin was administered every three weeks (q3W C AUC 5–6). To avoid bevacizumab heterogeneity in GOG 0262, only the data from the group that did not receive bevacizumab was included in the analysis.

**Fig. 2:**
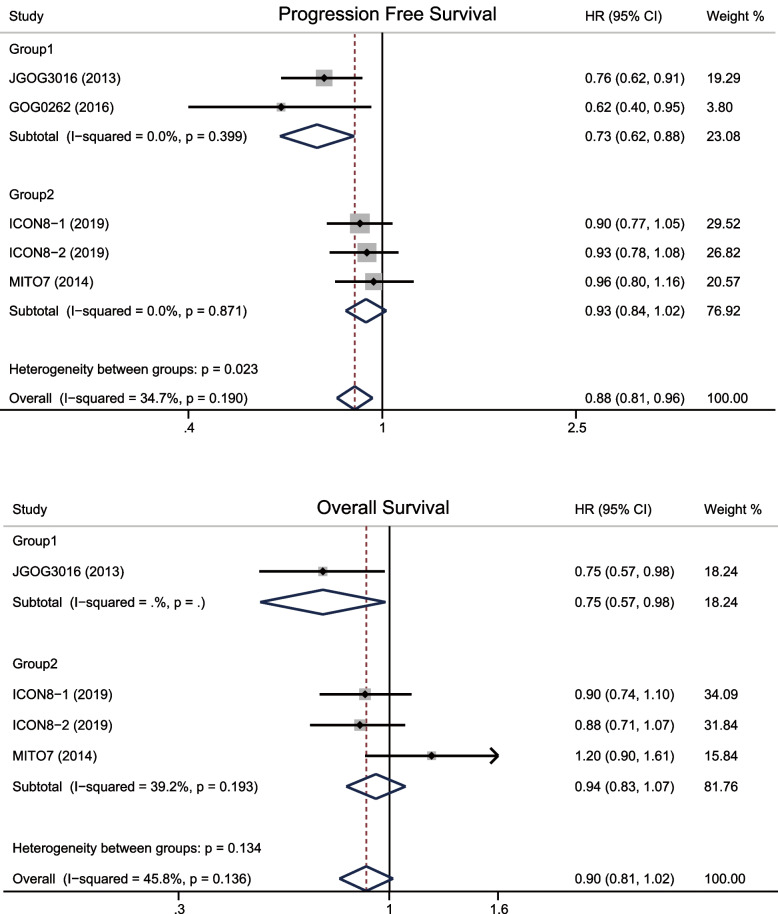
Forest plots for progression free survival and overall survival

**Fig. 3:**
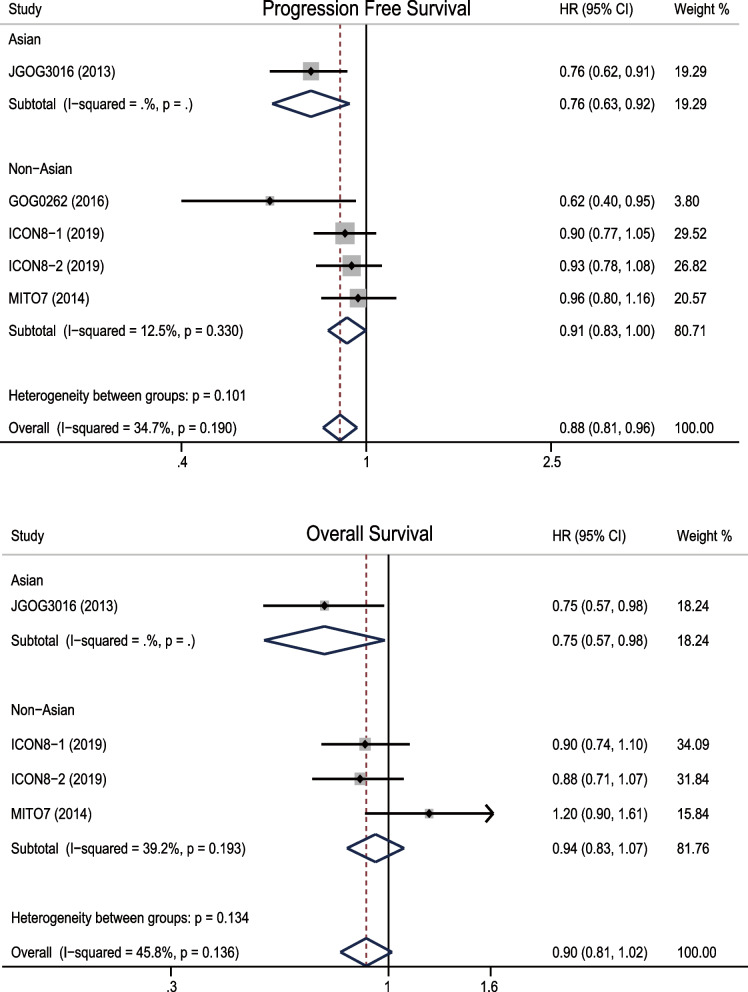
Forest plots for progression free survival and overall survival according to ethnics

In group 1, HR of PFS was 0.73 (95%CI 0.62–0.88; *p* = 0.001), HR of OS was 0.75 (95%CI 0.57–0.98; *p* = 0.037); in group 2, HR of PFS was 0.93 (95%CI 0.84–1.02; *p* = 0.118), HR of OS was 0.94 (95%CI 0.83–1.07; *p* = 0.371). In terms of all trials, dose-dense regimen could prolong PFS (HR 0.88, 95%CI 0.81–0.96; *p* = 0.002) and OS (HR 0.90, 95%CI 0.81–1.02; *p* = 0.09) (Fig. 2). The results of two groups of those trials were very heterogeneous, a subgroup analysis was performed based on ethnicity, which revealed that dose-dense regimen could significantly prolong PFS (HR 0.76, 95%CI 0.63–0.92; *p* = 0.005 vs HR 0.91, 95%CI 0.83–1.00; *p* = 0.046) and OS (HR 0.75, 95%CI 0.557–0.98; *p* = 0.037 vs HR 0.94, 95%CI 0.83–1.07; *p* = 0.371) in Asians (Fig. 3).

### Meta-analysis of toxicity

**Fig. 4:**
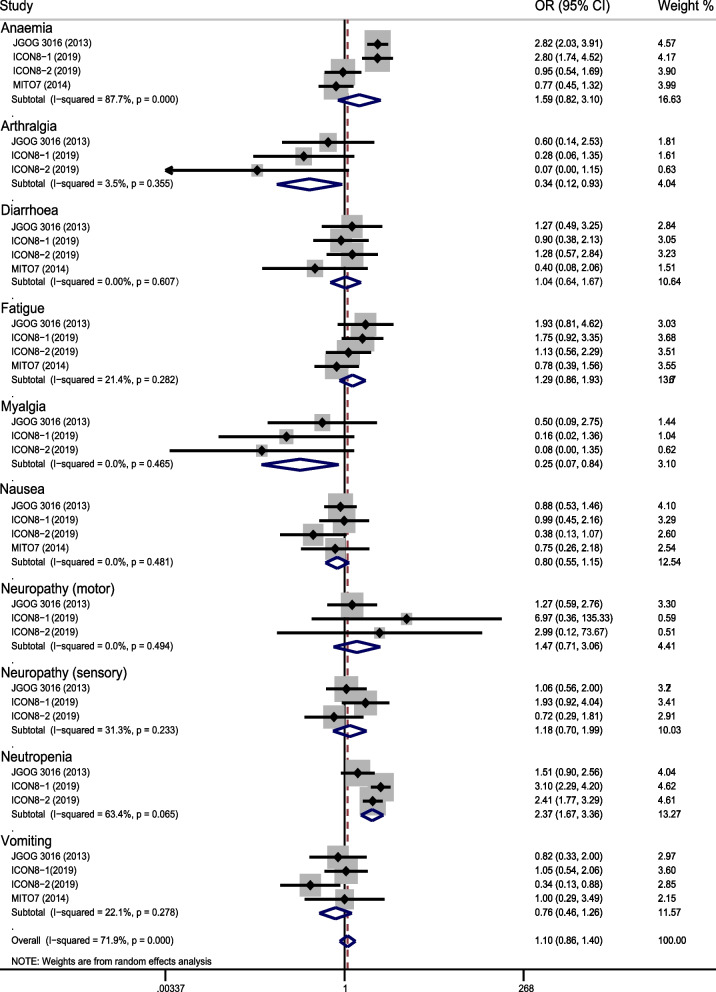
Forest plots for overall toxicity

**Fig. 5:**
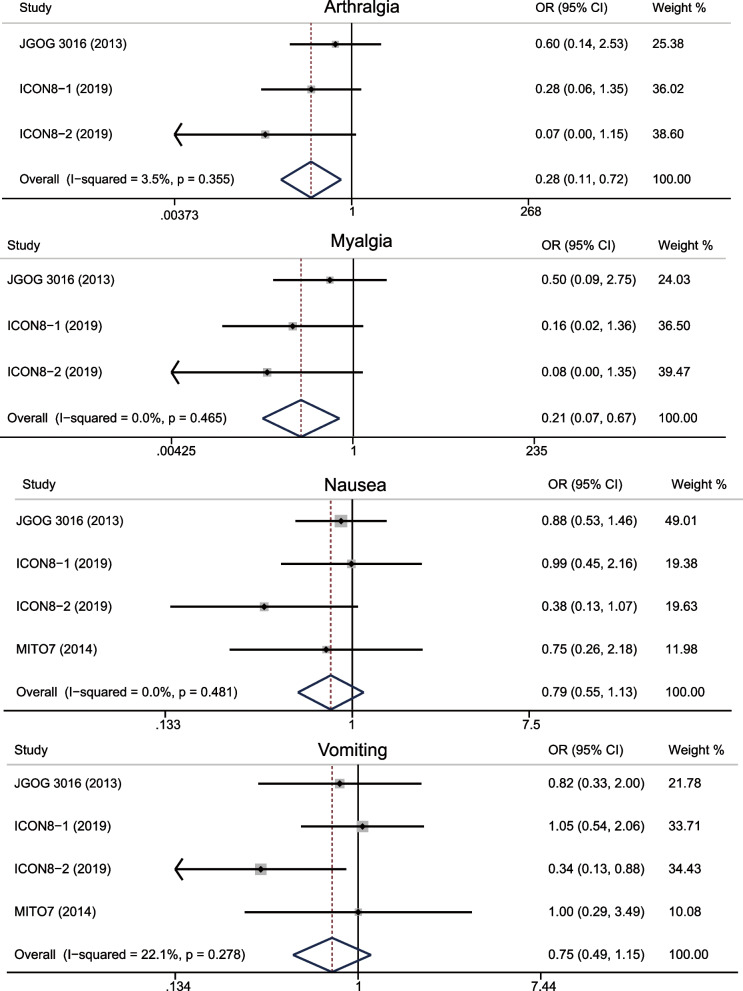
Forest plots for reduced toxicity

**Fig. 6:**
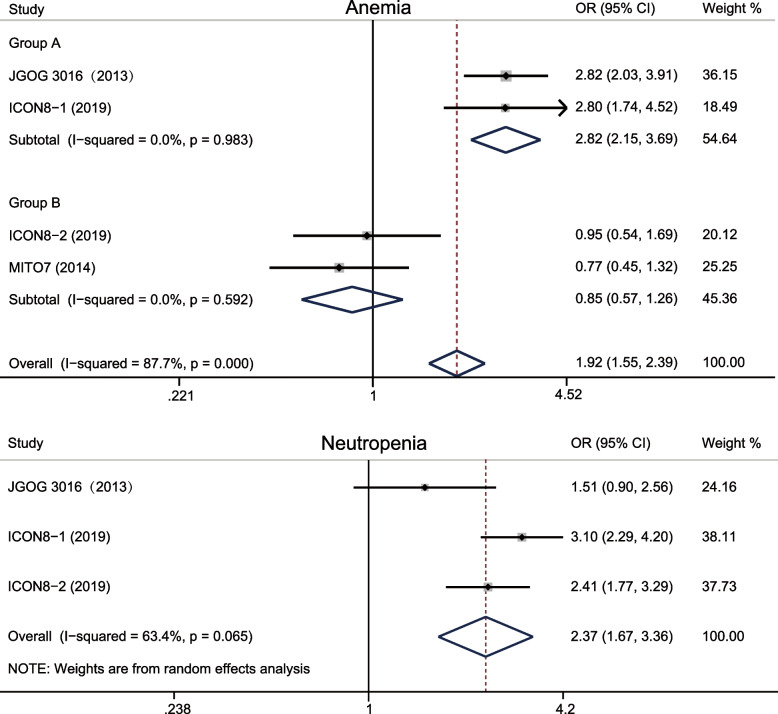
Forest plots for increased toxicity

**Fig. 7:**
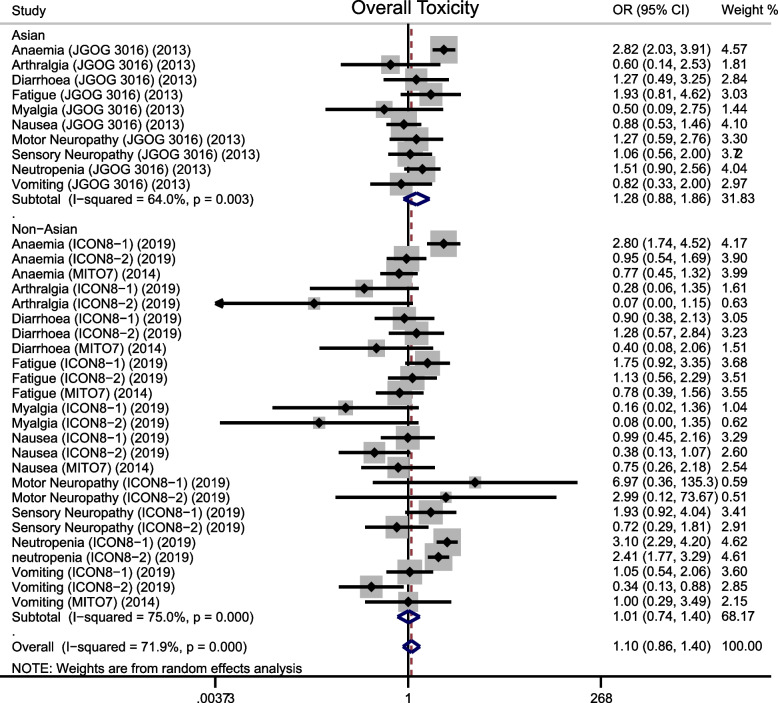
Forest plots for overall toxicity according to ethnics

Overall, dose-dense regimen could increase overall toxicity (OR = 1.102, 95%CI 0.864–1.405; *p* = 0.433) (Fig. 4), but it reduced toxicity of arthralgia (OR = 0.278, 95%CI 0.108–0.720; *p* = 0.008), myalgia (OR = 0.21, 95%CI 0.066–0.666; *p* = 0.008), nausea (OR = 0.788, 95%CI 0.548–1.134; *p* = 0.200), and vomiting (OR = 0.752, 95%CI 0.490–1.153; *p* = 0.191) (Fig. 5). It increased toxicity of anemia (OR = 1.924, 95%CI 1.548–2.391; *p* < 0.0001), neutropenia (OR = 2.372, 95%CI 1.674–3.361; *p* < 0.0001) (Fig. 6), diarrhea (OR = 1.027, 95%CI 0.641–1.644; *p* = 0.913), fatigue (OR = 1.290, 95%CI 0.906–1.836; *p* = 0.158), motor neuropathy (OR = 1.57, 95%CI 0.771–3.199; *p* = 0.214), sensory neuropathy (OR = 1.198, 95%CI 0.788–1.823; *p* = 0.398) (Figure S1). Subgroup analysis indicated that dose-dense regimen could significantly increase overall toxicity in Asians (OR = 1.28, 95%CI 0.877–1.858; *p* = 0.202) compared to non-Asians (OR = 1.02 95%CI 0.737–1.396; *p* = 0.929) (Fig. 7).

## Discussion

Paclitaxel combined with carboplatin chemotherapy regimen is an important method to improve the prognosis of advanced ovarian cancer. However, dose-dense regimen has been controversial in different clinical trials in recent years. This systematic review and meta-analysis tried to systematically review the RCTs of paclitaxel dose-dense regimen for ovarian cancer in recent years, and evaluate the efficacy and safety of paclitaxel dose-dense chemotherapy in primary epithelial ovarian cancer.

Based on the meta-analysis, dose-dense regimen revealed better PFS and OS, but it also increased overall toxicity. The following reasons might be able to explain the survival benefits. First, paclitaxel dose-density therapy could prolong the cumulative exposure time through multiple administrations in a short period. Emerging evidence had demonstrated that exposure time was a pivotal determinant of paclitaxel cytotoxic activity, and sufficient cytotoxicity could be exerted under relatively low blood concentration if exposure time were extended [13, 24, 25]. Second, dose-dense therapy delivered chemotherapy drugs weekly, which could shorten the interval time and increase the density of drug delivery [26]. Besides, the valley of paclitaxel blood concentration was higher than that of three-weekly therapy [27], and the cumulative dose of paclitaxel for dose-dense therapy was more than that of conventional three-weekly therapy (240 mg VS 175 mg) [25]. Last but not least, dose-dense regimen could kill more tumor cells in a shorter time, resulting in more tumor-infiltrating lymphocyte infiltration, and exerting therapeutic effects through immune responses [28, 29].

In terms of toxicity, the overall toxicity of dose-dense regimen increased slightly, mostly in blood toxicity, but there was also decreased toxicity such as vomiting. The following reasons might account for this situation, on the one hand, dose-dense regimen required routine blood tests every week, while conventional therapy every three weeks, so routine blood tests for three-weekly therapy showed that blood cells recover better; on the other hand, dose-dense regimen led to longer exposure times and shorter interval times, resulting in increased accumulation of paclitaxel, therefore, some side effects like anemia could increase [30].

According to the subgroup analysis results, the PFS and OS survival benefits of Asians from dose-dense regimen were higher than that in Europe and the United States. In addition, studies had shown that Asians were an independent factor in improving the survival rate of advanced ovarian cancer, it was believed that Asian women had better PFS in ovarian cancer than non-Asian women, ovarian cancer progressed more slowly in Asians, indicating that Asians were more suitable for weekly treatment [28]. The difference may be due to the following reasons.

For one thing, previous studies had revealed that there were significant differences in pharmacogenetics of paclitaxel drug metabolism between the United States and Japan, which resulted in differences in tumor chemotherapy outcomes [29]. For another thing, high-grade serous ovarian carcinoma was divided into four subtypes with various prognosis according to gene expression: C1/Mesenchymal, C2/Immunoreactive, C4/Differentiated, and C5/Proliferative [30], C1/Mesenchymal subtype treated with paclitaxel obtained survival benefit compared with non-paclitaxel treatment [31]. Histopathological analysis found that the median PFS of patients with mesenchymal transition (MT, a high-level) type receiving paclitaxel dose-dense regimen was significantly improved compared with the standard three-weekly treatment regimen (*p* = 0.01), suggesting that MT-type ovarian cancer patient might be suitable for the paclitaxel-dense regimen [15]. Therefore, the difference in treatment results between Europe, America and Japan might be attributed to the diverse pathological distribution. Japan might have had more mesenchymal transition types, leading to a better prognosis. In Europe and America, the uneven distribution of pathological subtypes might lead to various results in different trials [32].

Meanwhile, toxicity in Japan was higher than in Europe and America [33]. Previous studies demonstrated that among lung cancer and breast cancer patients treated with paclitaxel, the toxicity in Asians was significantly higher than that in Caucasians. This might be due to the racial difference of some allelic variants encoding drug-metabolizing enzymes, which led to differences in metabolic enzyme functions and thus altered pharmacokinetics, contributing to slightly greater toxicity in Asians [34, 35].

Based on these randomized controlled studies, we concluded that paclitaxel dose-density therapy was more suitable for advanced ovarian cancer in the following populations: 1. Patients with high-grade serous ovarian cancer, especially Mesenchymal Transitions type [14]; 2. Patients with residual lesions greater than 1 cm after surgery [14]; 3. Patients who cannot receive bevacizumab [11]; 4. Elderly people over 70 years old with multiple tumor complications and dysfunction [19]; 5. Patients who are in poor health and cannot tolerate a large dose of chemotherapy [4].

There were some limitations in our research. First, there was only one RCT in Asia (JGOG3016), and the number of patients was not as large as in Europe. Second, GOG0262 had no OS data for analysis, which reduced the credibility of the analysis results. Besides, since dose-dense regimen was still under investigation and had not yet established a well-accepted regimen, there was heterogeneity in carboplatin dosage and schedule among the included trials, which might lead to bias in the comparison of the two regimens. Last, although a strict search formula had been done and searched in multiple databases, it could not be ruled out that some grey documents, conference abstracts, etc. were missed.

## Conclusion

In conclusion, our study systematically reviewed the randomized controlled trials of paclitaxel plus carboplatin for dose-dense regimen and conventional three-weekly therapy. Some RCTs have revealed that dose-dense regimen could prolong PFS and OS in advanced ovarian cancer patients, but some studies indicated that the dose-dense regimen did not bring significant benefits. This meta-analysis demonstrated that the dose-dense regimen could prolong PFS and OS, but it also increased the overall toxicity. However, the toxicity of arthralgia, myalgia, nausea and vomiting had decreased. Subgroup analysis suggested Asians might benefit from dose-dense regimen in both PFS and OS with slightly increased overall toxicity, which needs to be further investigated in future clinical trials in Asians.

## Supplementary Information

**Additional file 1: Figure S1.** Forest plots for increased toxicity.

## Data Availability

All datasets generated for this study are included in the article/Supplementary Material.

## Abbreviations

AUC: Area under the curve
RCT: Randomized controlled trials
PFS: Progression free survival
OS: Overall survival
OR: Odds ratio
HR: Hazard ratio
MT: Mesenchymal transition
