# Supplementary figures and images for: Dose-dense regimen versus conventional three-weekly paclitaxel combination with carboplatin chemotherapy in first-line ovarian cancer treatment: a systematic review and meta-analysis
Source: J Ovarian Res. 2023 Jul 10;16:136. doi: 10.1186/s13048-023-01216-z (PMC10331960; doi:10.1186/s13048-023-01216-z)

Figure S1: Forest plots for increased toxicity

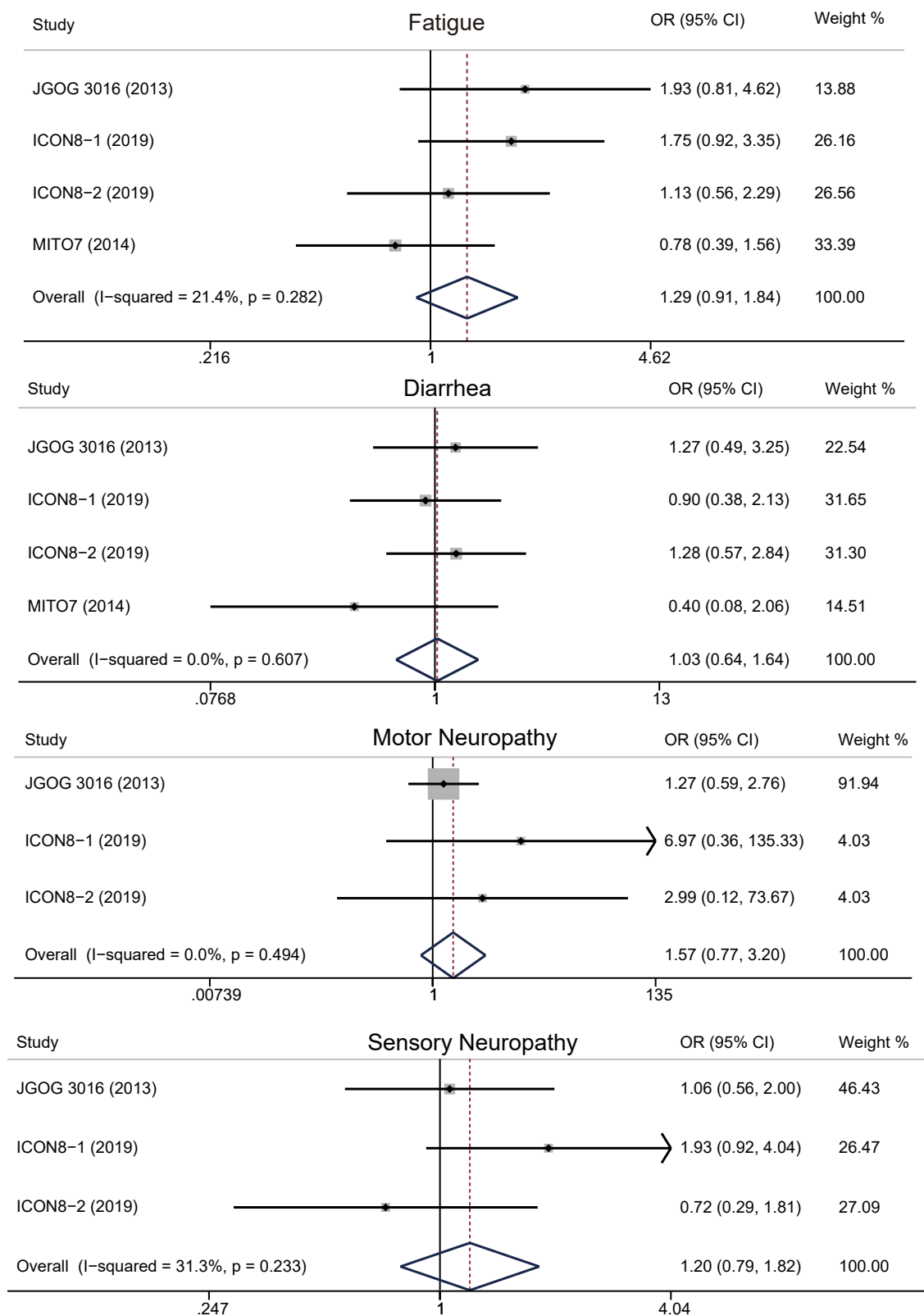

Supplement: Supplementary file 1 — Additional file 1: Figure S1. Forest plots for increased toxicity. [file 13048_2023_1216_MOESM1_ESM.pdf]
